# Bone morphology and physical characteristics of the pro-cyclist hip joint

**DOI:** 10.1007/s00264-024-06196-3

**Published:** 2024-05-03

**Authors:** Shunsuke Akiho, Ryuki Hashida, Yoshihiko Tagawa, Akira Maeyama, Koichi Kinoshita, Kazuki Kanazawa, Hiroo Matsuse, Masafumi Hara, Takuaki Yamamoto

**Affiliations:** 1 Department of Orthopaedic Surgery, Fukuoka University Faculty of Medicine, 7-45-1 Nanakuma, Jonan-Ku, Fukuoka, 810-0180 Japan; 2 Orthopaedic Surgery, Fukuoka Seisyukai Hospital, 4-11-8 Chojyabarunishi, Kasuya-Machi, Kasuya-Gun, Fukuoka, 811-2316 Japan; 3 Division of Rehabilitation, Kurume University Hospital, 67 Asahimachi, Kurume, 830-0011 Japan; 4 Department of Orthopaedic, Kurume University School of Medicine, 67 Asahimachi, Kurume, 830-0011 Japan; 5 Orthopaedic Surgery, Hisatsune Hospital, 152-1 Tadomiushimaru, Sime-Machi, Kasuya-Gun, Fukuoka, 811-2204 Japan

**Keywords:** Professional cyclists, Acetabular dysplasia, Hip internal rotation, Metabolic efficiency, Simulation, Range of motion

## Abstract

**Purpose:**

This study aimed to investigate the radiographic findings for the hip joint and hip range of motion in professional cyclists, and to determine their bone morphology and physical characteristics. The effects of physical characteristics on athletic performance were examined in terms of metabolic efficiency using simulation analysis.

**Methods:**

We performed a case–control research study on 22 hips in 11 male professional cyclists (average age 28.5, height 1.73 m, weight 77.6 kg). Thirty hips in 15 healthy male volunteers were selected as controls. As radiographic evaluations, acetabular dysplasia was assessed on standardized radiographs. During physical evaluations, the hip range of motion was examined. We used simulation analysis to investigate the metabolic efficiency in the different cycling forms.

**Results:**

The radiographic evaluations showed a significant difference in the incidence of acetabular dysplasia (p = 0.01): 59% (13/22 hips) in the pro-cyclist group versus 10% (3/30 hips) in the control group. The physical evaluations revealed significant differences in the hip internal rotation angle (p = 0.01), with greater ranges of internal rotation in the pro-cyclist group versus the control group. The simulation analyses showed that metabolism was reduced in the cycling form with hip internal rotation, especially in the lower extremities.

**Conclusions:**

Pro-cyclists showed a high frequency of acetabular dysplasia and superior hip internal rotation. According to the cycling model analyses, hip internal rotation allowed pedaling with reduced metabolic power.

## Introduction

Low back pain and knee pain are the most common overuse injuries in track cycling [1, 2]. The incidence of low back and knee pain is reported to be 45% and 23%, respectively [1]. Regarding low back pain, several reports have demonstrated relationships with spinal alignment [3] and mobility of the pelvis and lower extremities [4]. For the hip joint, the incidence of hip pain in elite cyclists who trained for an average of ten hours per week was reported to be approximately 18% [5]. However, the pathogenesis of hip pain has not been clarified [6]. The morphological characteristics of the hip joint in professional cyclists and their impact on competitive performance are not well understood. Furthermore, the impact of hip joint mobility on cycling track performance has rarely been evaluated.

Against this background, the purpose of this study was to evaluate hip joint radiographic findings in professional cyclists, with a particular focus on acetabular dysplasia and the hip range of motion. We investigated the relationship between bone morphology of the acetabulum and hip joint mobility and its impact on performance in cycling track events in detail. In addition, we conducted analyses using model simulations to aid in the recognition of competitive characteristics.

## Materials and methods

### Ethical approval

This study was conducted following the Declaration of Helsinki and was approved by the Ethics Committee. All the subjects approved participation in this study. Informed consent was obtained from all the subjects.

### Background characteristics

We performed a case–control study on 22 hips in 11 male professional cyclists. The mean age was 28.5 years (range, 22–43 years), mean height was 1.73 m (range, 1.64–1.82 m), mean weight was 77.6 kg (range, 65–90 kg), and mean body mass index (BMI) was 25.8 kg/m2 (range, 21.0–30.1 kg/m2). All the cyclists were relatively young athletes with a mean competition period of 8.1 years (range, 2–15 years). Thirty hips in 15 healthy male volunteers were selected as the controls. The mean age of the volunteers was 25.9 years (range, 23–35 years), mean height was 1.73 m (range, 1.67–1.79 m), mean weight was 72.1 kg (range, 62–85 kg), and mean BMI was 24.4 kg/m2 (range, 20.7–28.1 kg/m2).

### Radiographic evaluations

**Fig. 1:**
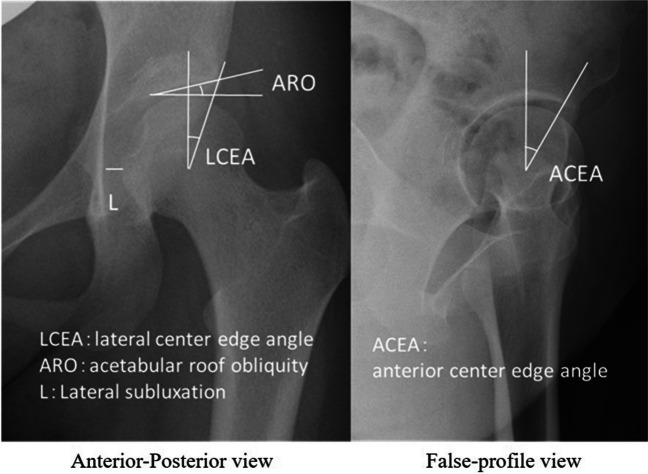
Radiographic evaluations

In this study, acetabular dysplasia was assessed on standardized radiography. To evaluate this condition, we measured the lateral centre–edge angle (LCEA) [7]. An LCEA less than 20° was defined as indicative of acetabular dysplasia [8, 9]. Furthermore, we measured acetabular roof obliquity (ARO) [10] and lateral subluxation [7] from the anteroposterior view, and the anterior centre–edge angle (ACEA) [11, 12] from the false-profile view (Fig. 1).

The measurements were performed by two orthopedic surgeons who were blinded to the clinical results. The same observers reviewed the radiographs three times on different days, and the mean values were calculated. The measurements were evaluated for interobserver reliability. The intraclass correlation coefficient ICC (2.3) for these measurements was 0.92.

### Physical evaluations

The hip range of motion was evaluated. The flexion, abduction, adduction, internal rotation, and external rotation angles were measured in the supine position, and the extension angle was measured in the prone position. The measurements were performed by two physical therapists with more than ten years of experience who were blinded to the clinical results. Each observer reviewed the range of motion once on the same day, and the mean values were calculated. The measurements were analyzed for interobserver reliability. The intraclass correlation coefficient ICC (2.1) for these measurements was 0.89.

### Simulation analysis

To evaluate the effect of acetabular dysplasia on cycling, we performed motion analyses using a kinematic cycling model. The simulation analyses were carried out using the AnyBody software ver.7.3.2 (AnyBody Technology A/S, Aalborg, Denmark) musculoskeletal modeling system. The human body model in the software is based on the median European male body and has more than 200 bones and 1,000 muscles. The analysis is based on inverse dynamics, and calculates muscle forces, moments, and other data for given motions. The model configuration can be selected according to the intended use.

The full-body model of the AnyBody AMMR (AnyBody Managed Model Repository) BikeModel was used as the bicycle ergometer cycling motion model. The Hill-type muscle model consisting of tendon-elastic elements, contractile elements, and parallel-elastic elements [13] was introduced to analyze biomechanics and movement in cycling. The muscle recruitment of the model was obtained by a combination of the min/max criterion that minimizes the maximum muscle activity to delay fatigue [14] and the quadratic criterion that minimizes the series of quadratic terms composing the energy-related cost function for calculating the inverse dynamics of the musculoskeletal system.

For the study, we assumed a height of 1.73 m and weight of 78 kg, resembling the average body shape for the professional cyclists belonging to Keirin. In consideration of the well-trained body, fat percentage of 10%, muscle strength of 1.5 times normal. Hip position of 0.72 m high from the crankshaft. Deep forward flexion of the upper body and forward movement of the hip joints on the saddle are effective for reducing aerodynamic drag at high speed [15]. The simulations were performed with a cadence of 150 rpm and a mechanical load of 1,200 W, incorporating the characteristics of bicycle bodywork used by professional cyclists.

**Fig.2:**
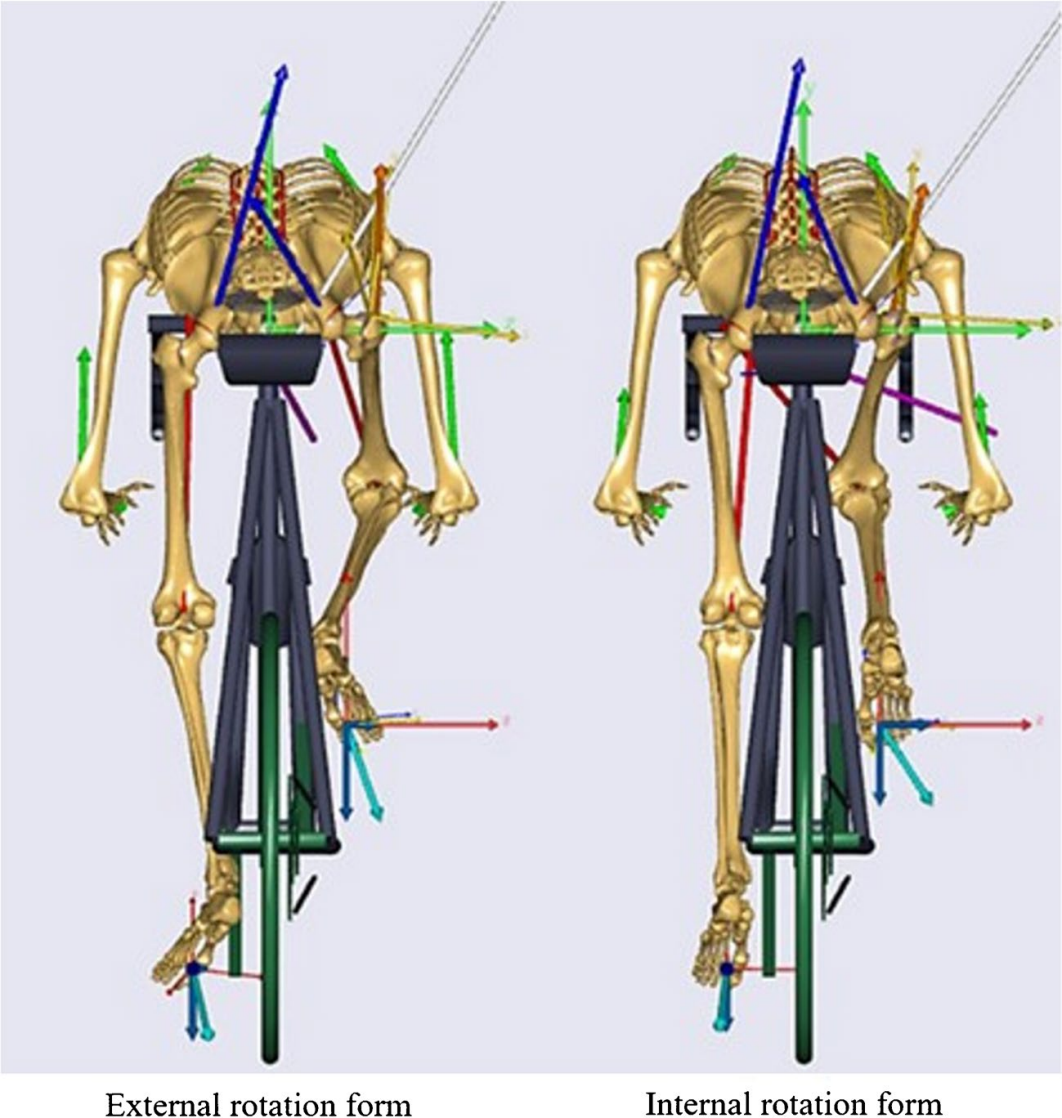
Comparison of metabolic power for different forms of cycling athletes

The AnyBody software can determine muscle metabolic power using Umberger's detailed metabolic model [16]. We compared the metabolic power of pedaling with the knee joint facing outward (external rotation form) and pedaling with the hip joint internally rotated and the knee joint facing inward (internal rotation form) as the forms of cycling athletes (Fig. 2). In addition, the metabolic power of each lower limb muscle was evaluated in both the external and internal rotation forms.

### Statistical analysis

Radiographic and physical evaluations were compared between the pro-cyclist and control group. To evaluate the effect of acetabular dysplasia on physical characteristics, the pro-cyclist group was divided into two subgroups: the acetabular dysplasia subgroup (13 hips) and the non-dysplasia subgroup (11 hips), and physical evaluations were compared between the two subgroups.

The Mann–Whitney U test and chi-square test were used to compare the data for the pro-cyclist (22 hips) and control (30 hips) groups. To evaluate the effects of acetabular dysplasia on the physical characteristics, the 22 pro-cyclist hips were divided into two subgroups: acetabular dysplasia group with LCEA < 20° (13 hips) and non-dysplasia group with LCEA ≥ 20° (9 hips). The Mann–Whitney U test was used for comparisons between the data for the two subgroups. Statistical significance was defined as p < 0.05. All the statistical analyses were performed using JMP Pro15 (SAS Institute Inc., Cary, NC, USA).

## Results

### Bone morphology of the pro-cyclist hip joint: Evaluation by X-ray parameters

**Fig. 3:**
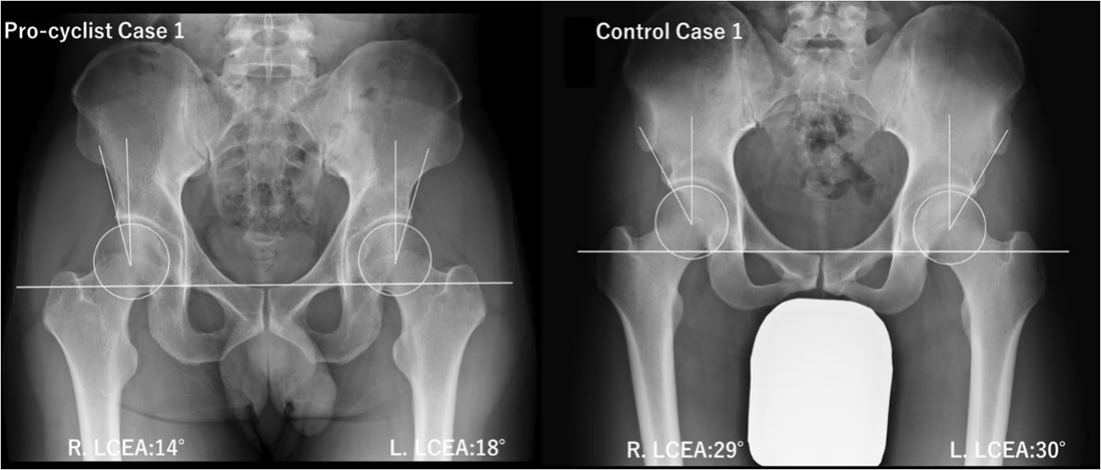
The anteroposterior radiographs and corresponding LCEA data for Pro-cyclist Case 1 and Control Case 1

The morphological characteristics of the hips of 11 pro-cyclists were evaluated using standardized radiographs, with the measurement results presented in Table 1. Of all the cyclists, eight out of 11 had acetabular dysplasia, with an LCEA less than 20°. The ACEA in cyclists with acetabular dysplasia was less than 20° except Pro-cyclist Case 7. The anteroposterior radiographs and corresponding LCEA data for pro-cyclist Case 1 and control Case 1 are shown in Fig. 3. Pro-cyclist Case 1 exhibited acetabular dysplasia bilaterally, with an LCEA of 14° on the right and 18° on the left. The ARO was 15° on the right and 11° on the left, indicating a tilt of the acetabulum. The ACEA was 17° on the right and 19° on the left, confirming a deficiency in anterior coverage.

**Table 1:** Morphological hip parameters of pro-cyclists measured values

	LCEA (deg.)		ARO (deg.)		Lateral subluxation (mm)		ACEA (deg.)	
Case number	R	L	R	L	R	L	R	L
Pro-cyclist 1	14	18	15	11	10	9	17	19
Pro-cyclist 2	20	18	6	7	9	9	23	18
Pro-cyclist 3	28	26	0	0	10	9	28	27
Pro-cyclist 4	19	17	7	6	10	10	13	9
Pro-cyclist 5	18	16	3	6	9	10	15	14
Pro-cyclist 6	18	19	8	8	8	8	18	17
Pro-cyclist 7	23	17	5	7	10	9	30	22
Pro-cyclist 8	26	23	2	5	9	9	32	33
Pro-cyclist 9	24	18	1	3	10	10	13	19
Pro-cyclist 10	27	23	-1	4	9	9	25	30
Pro-cyclist 11	17	17	7	6	9	8	15	19
Mean	21.3	19.3	4.8	5.7	9.4	9.1	20.8	20.6
Control 1	29	30	0	0	9	9	42	38
Dysplasia hip (LCEA < 20)								

Abbreviations: *LCEA* lateral center–edge angle, *ARO* acetabular roof obliquity, *ACEA* anterior center–edge angle

### Findings for the pro-cyclist group versus the control group

The background characteristics, radiographic findings, and physical examination findings for the two groups are shown in Table 2. There were no significant differences in age, height, weight, or BMI between the two groups. Radiographic evaluations revealed a significant difference in the incidence of acetabular dysplasia between the pro-cyclist group and the control group (Pro-cyclist group; 59% [13/22 hips], control group; 10% [3/30 hips]) (p = 0.01). The physical evaluations revealed significant differences in the hip flexion angle (p = 0.04) and internal rotation angle (p = 0.01), with greater ranges of flexion and internal rotation in the pro-cyclist group than in the control group.

**Table 2:** Background characteristics, radiographic findings, and physical examination findings in the pro-cyclist and control groups

	Pro-cyclist	Control	P-value
Background			
Age (year)	28.5 ± 6.3	25.8 ± 3.3	0.388
Height (m)	1.73 ± 0.06	1.73 ± 0.03	0.515
Weight (kg)	77.6 ± 8.0	72.1 ± 6.4	0.077
Body mass index (kg/m^2^)	25.8 ± 2.4	24.2 ± 2.1	0.108
Radiographic evaluations			
LCEA (deg.)	20.3 ± 4.0	26.1 ± 4.7	< 0.001*
ARO (deg.)	5.3 ± 3.7	3.4 ± 3.5	< 0.001*
Lateral subluxation (mm)	9.2 ± 2.1	8.8 ± 2.7	0.233
ACEA (deg.)	20.7 ± 6.9	31.6 ± 7.1	< 0.001*
Acetabular dysplasia (n, %)	13, 59%	3, 10%	0.011*
Hip Range of Motion			
Flexion (deg.)	124.5 ± 5.1	121.2 ± 7.1	0.038*
Extension (deg.)	9.8 ± 2.9	9.6 ± 2.4	0.549
Abduction (deg.)	39.5 ± 7.7	42.5 ± 6.7	0.092
Adduction (deg.)	12.0 ± 5.3	13.1 ± 4.4	0.579
Internal rotation (deg.)	30.9 ± 7.2	24.6 ± 8.1	0.006*
External rotation (deg.)	42.5 ± 8.4	40.2 ± 5.9	0.324

*Significant difference (p < 0.05)

Abbreviations: *LCEA* lateral center–edge angle, *ARO* acetabular roof obliquity, *ACEA* anterior center–edge angle

### Findings for the acetabular dysplasia subgroup versus the non-dysplasia subgroup

Univariate analyses of the findings for the 22 pro-cyclist hips after division into the acetabular dysplasia subgroup (13 hips) and non-dysplasia subgroup (9 hips) are shown in Table 3. Hip internal rotation differed significantly between the two subgroups (p = 0.02), with a greater range of internal rotation in the acetabular dysplasia group versus the non-dysplasia group.

**Table 3:** Comparisons of 22 hips among 11 professional cyclists after being divided into dysplasia (LCEA < 20°; n = 13) and non-dysplasia (LCEA ≥ 20°; n = 9) subgroups

	Acetabular dysplasia	Non-dysplasia	*P*-value
Mean LCEA (deg.)	17.4	24.4	
Hip Range of Motion			
Flexion (deg.)	125.4 ± 4.7	123.3 ± 5.6	0.366
Extension (deg.)	10.4 ± 2.5	8.9 ± 3.3	0.239
Abduction (deg.)	40.4 ± 8.9	40.7 ± 7.2	0.764
Adduction (deg.)	11.8 ± 4.6	11.4 ± 6.2	0.764
Internal rotation (deg.)	33.8 ± 6.2	26.7 ± 6.6	0.021*
External rotation (deg.)	41.2 ± 6.8	44.4 ± 10.4	0.342

*Significant difference (p < 0.05)

Abbreviations: *LCEA* lateral center–edge angle

### Simulation analysis

**Fig.4:**
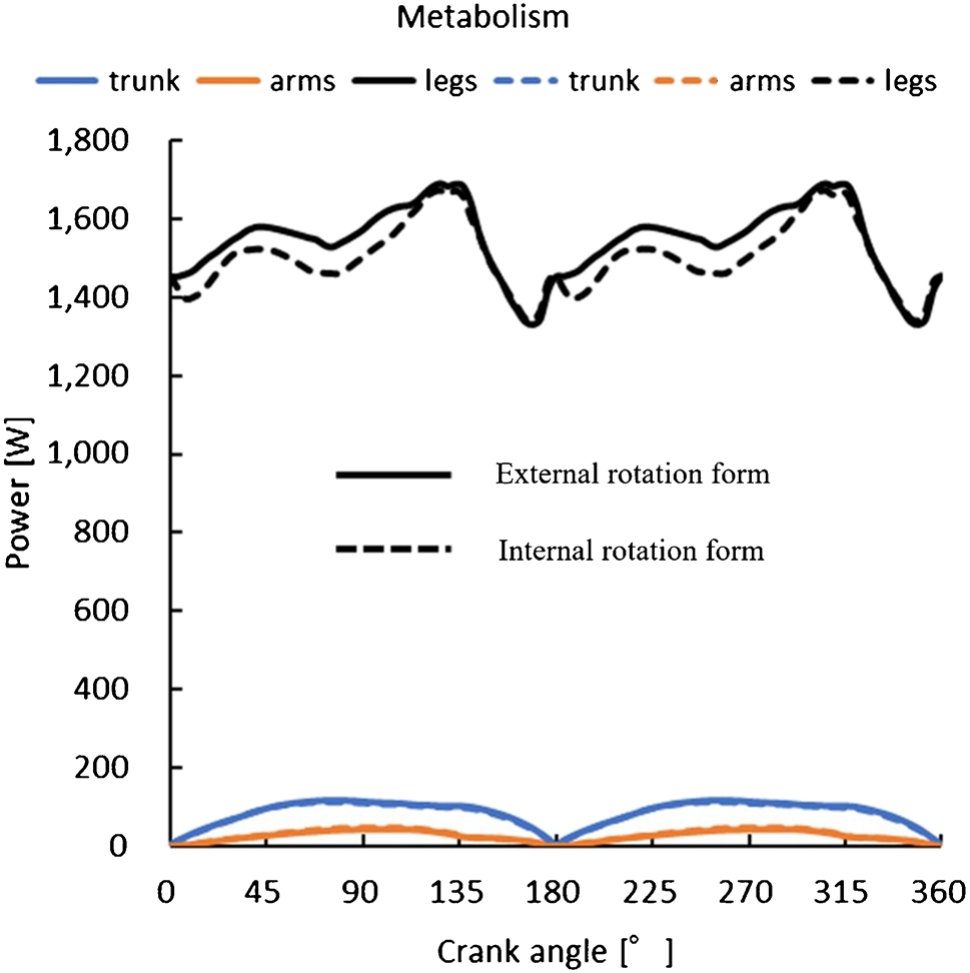
Differences in form and metabolic energy

**Fig.5:**
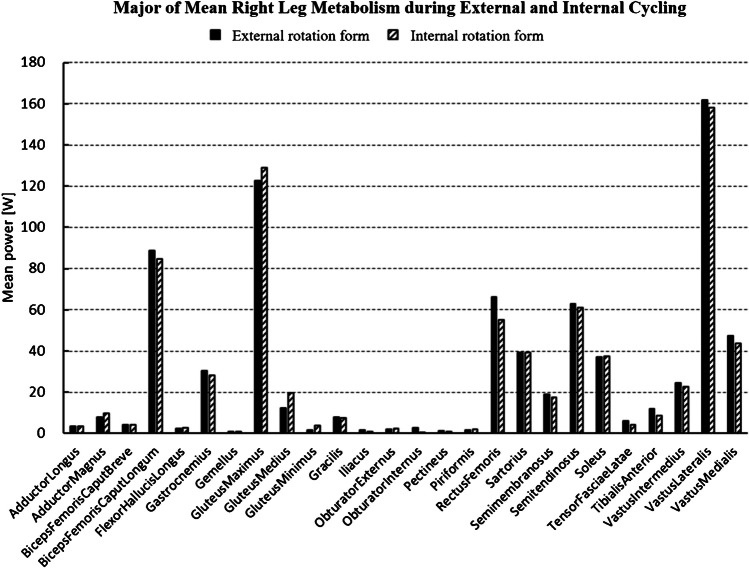
Differences in form and metabolic power of each lower limb muscle

In this simulation analysis, we compared the metabolic efficiency of different cycling forms. Under the previously mentioned settings, the total systemic metabolism per cycle averaged 1,655 W in the external rotation form and 1,613 W in the internal rotation form. We found that metabolism was reduced in the cycling form with hip internal rotation, especially in the lower extremities (Fig. 4). Upon evaluating individual lower limb muscles, it was noted that the quadriceps, vastus medialis, vastus lateralis, and all hamstring muscles exhibited reduced metabolic activity in the internal rotation form. Notably, the metabolism of the rectus femoris muscle was significantly suppressed. Conversely, the metabolism of the gluteus maximus, gluteus medius, and gluteus minimus muscles was increased (Fig. 5).

## Discussion

In this study, we found that acetabular dysplasia was frequently observed in professional cyclists. Moreover, the athletes with acetabular dysplasia had a superior hip internal rotation. Simulation results suggested that a systemic metabolism was reduced in the cycling form with hip internal rotation. Acetabular dysplasia, which allows smooth internal hip rotation, may be an advantageous physical characteristic in track cycling.

The mean LCEA for the 22 pro-cyclist hips was 20.3°, and 59% (13 hips) had acetabular dysplasia. In terms of the prevalence of acetabular dysplasia, Lau et al. reported 2.1% in 999 Hong Kong residents [17], Inoue et al. reported 5.1% in 820 Japanese residents [18], and Jacobsen et al. reported 6.7% in 1,352 Danish residents [19]. Compared with the findings in other reports [20–22], the frequency of occurrence was higher in our pro-cyclists.

Among other sports athletes, it was reported that the risk of injury increases with decreased hip internal rotation [23] and that hip range of motion affects performance [24]. For example, a report investigating the relationship between baseball pitching form and ball speed has indicated that a decrease in hip flexion and an increase in abduction lead to a decrease in ball velocity [24]. However, there are no reports investigating the hip range of motion in cyclists. In the present study, the pro-cyclists had better hip flexion and internal rotation than the healthy control subjects. Moreover, the pro-cyclists with LCEA < 20° had a better range of internal rotation than the pro-cyclists with LCEA ≥ 20°. A shallow hip joint with small acetabular coverage of the LCEA can easily avoid collision with the femur. Therefore, it was presumed that the hip joint internal rotation movement would become smoother.

Various biomechanical studies have been conducted on bicycle athletes [25, 26]. Metabolic efficiency is the main index used to evaluate athlete-related factors for cycling performance [27]. In the present study, we investigated the effect of hip internal rotation on competition performance by evaluating the metabolic efficiency of different cycling forms. The simulation analyses showed that metabolism of the rectus femoris muscle was reduced in the internal rotation form, while metabolism of the gluteus medius muscle group was slightly increased. Taken together, these findings suggest that the internal rotation form suppresses metabolism during cycling compared to the external rotation form, thereby enhancing metabolic efficiency.

The reason why the internal rotation form reduces energy metabolism during cycling has not been determined, but the following reasons can be considered. According to the study by Silva et al., it has been shown that the rectus femoris muscle is involved primarily in hip flexion and knee extension during pedaling [28]. Baldon et al. noted the contribution of the gluteal muscles to the increase in hip internal rotation [29]. From an anatomical perspective, when the hip joint rotates internally, the greater trochanter, which is the attachment point of the gluteus medius, moves anteriorly. Consequently, the anterior fibers of the gluteus medius, which flexes the hip joint, become almost equal to the direction of contraction of the rectus femoris muscle. This alignment facilitates the exertion of relatively strong muscular force. In essence, the gluteus medius and rectus femoris muscles can work together to exert force. This interaction may also explain the increased metabolic efficiency of the rectus femoris muscle. The gluteus medius is an important stabilizer of the hip joint [30]. It has been reported that the gluteus medius muscle consists of anterior, middle, and posterior fibres, each with distinct functional characteristics [31]. However, the role of the gluteus muscle during deep hip flexion, such as during pedaling, remains to be elucidated. Further research is needed.

The results of this study indicate that not only the rectus femoris but also the hamstring muscles, which serve as hip extensors, exhibit decreased energy consumption in the internal rotation form. This improved metabolic efficiency in the extensor muscles is presumed to be a result of their interaction with the gluteal muscles, particularly the gluteus maximus [29]. Additionally, Wang et al. reported that fatigue in the hamstrings and vastus medialis significantly affects cycling performance [32]. Particularly at high speeds, the activity of the hamstring muscles is increased. These muscles are known to be rich in slow-twitch fibres which have high fatigue resistance and recovery capacity. Consequently, athletes with an easier internal rotation of the hip may efficiently utilize these muscles, reducing fatigue while conserving energy. This mechanism could be a contributing factor for athletes to maintain high performance in long-distance or high-intensity cycling.

Simulation techniques are useful for predicting internal characteristics such as muscle strength and metabolic power. The BikeModel, applied in this study, was validated under normal cycling conditions [33]. Simulation results closely matched experimental data regarding the relationship between variations in crank mechanical load and oxygen uptake, pedal rate and oxygen uptake, as well as the correlation between mechanical load changes and knee joint force. This strong correlation confirms the validity and reliability of using simulation methods for cycling analysis.

The present study has several limitations. First, the number of research subjects was small. A post-hoc analysis revealed an effect size of 0.53. Future studies involving a greater number of cases are required. Second, the knee joint movement in the simulation model is restricted to flexion and extension. Thus, it is necessary to examine this issue based on actual measurement data.

## Conclusions

We investigated the relationship between acetabular dysplasia and hip internal rotation and the impact of acetabular dysplasia on metabolism in the simulation cycling model. Professional cyclists showed a high frequency of acetabular dysplasia and superior hip internal rotation. Analyses of cycling models revealed that this internal rotation of the hip facilitates pedaling with reduced metabolic power demand.
